# Special Issue “Stem Cell Biology & Regenerative Medicine”

**DOI:** 10.3390/ijms241612855

**Published:** 2023-08-16

**Authors:** Rivka Ofir

**Affiliations:** BGU-iPSC Core Facility, The Regenerative Medicine & Stem Cell (RMSC) Research Center, Ben Gurion University of the Negev, Israel and Desert & Dead Sea R&D, Central Arava Branch, Be’er Sheva 84105, Israel; rivir@bgu.ac.il

More than 50% of pre-clinical studies fail despite a long and expensive journey of drug discovery using animal models [1]. Currently, there is a human-specific replacement: human patient induced pluripotent stem cells (hiPSCs)/human embryonic stem cells (hESCs) and their counterpart corrected cells (using Crispr methodology), which enable the establishment of disease-in-a-dish models encompassing patients’ genetics, which were eluted in this issue. This Editorial review contains three parts: cellular models, three-dimensional models and reviews.

Piera Trionfini et al. describe how allogeneic iPSCs and hypoimmunogenic iPSCs serve as new cost-effective and off-the-shelf opportunities for cell therapy in liver diseases. They achieved this goal by employing the CRISPR/Cas9 (clustered regularly interspaced short palindromic repeats/CRISPR associated protein 9) system to delete the β2-Microglobulin (*B2M*) and the Class II Major Histocompatibility Complex Transactivator (*CIITA*) genes [2]. LU PAN et al. employ a model of neural progenitor cell (NPCs) migration assay to answer the unsolved problem of the impacts of sevoflurane anesthesia on the impairment of NPC migration and, as such, on cognitive function. They were able to show that a sevoflurane-induced decrease in NPC migration is dependent on cyclophilin D (CypD) levels [3].

Seongjun SO et al. describe their methodology to generate corneal endothelial cells (CECs) from induced pluripotent stem cells (iPSCs) and suggest that these CECs can be a promising cellular resource for the treatment of corneal endothelial dysfunction (CED) cases [4]. Anna Klepikova et al. compared the productivity, the differentiation trajectories and the characteristics of iMacs generated using two widely used protocols: (1) Based on the formation of embryoid bodies and the induction of myeloid differentiation by interleukin-3 and macrophage colony-stimulating factor, and (2) utilizing multiple exogenous factors. Their results are very important for the field of iMac-based cell therapy, as they clearly showed that iMac characteristics depend on the type of differentiation protocol [5].

Yu Mi Park et al. investigated the effects of the transplantation of human arginine decarboxylase (hADC) murine brain-derived neural precursor cells (mNPCs) in a spinal cord injury (SCI) mouse model. They showed that locomotor and bladder function were both rehabilitated. These results showed that the transfection of the hADC gene exerts protective effects after injury in mNPCs, providing an empirical basis for gene therapy as a curative SCI treatment option [6].

Amelia Cataldi et al. studied how the mineralization of pulp mesenchymal stem cells (DPSCs, dental pulp stem cells) is affected by inducible NOS (iNOS) upregulation. The interest in this topic is related to the fact that dental pulp inflammation results from the invasion of dentin by pathogenic bacteria and involves excessive NO production. Their cellular model enabled them to screen for iNOS selective inhibition, suggesting that it can be considered an innovative therapeutic strategy to counteract inflammation and to promote the regeneration of the dentin–pulp complex [7].

Tawakalitu Okikiola Waheed et al. address the observation that mesenchymal stem/stromal cells (MSC) possess proliferation and differentiation potential, thus facilitating regenerative processes. However, the regenerative capacity of MSC might be impaired by oxidative stress: short- and long-term oxidative stress can be mimicked by exposure to H_2_O_2_ with or without glucose oxidase (GOx), respectively. It emerged that prolonged GOx-induced exposure to H_2_O_2_ allows for better cellular adaptation; however, the mechanism to explain the difference between short- and long-term adaptation is not clear [8]. Tereza Souralova et al. describe current good manufacturing practice (cGMP) that has to be implemented in the transport of embryos, the derivation of the inner cell mass to xeno-free, feeder-free and defined hESC culture, and cell freezing. They developed and validated a system for the manufacture of xeno-free and feeder-free clinical-grade hESC lines that present high-quality starting material suitable for cell therapy according to cGMP [9].

Eléanor Luce et al. demonstrate that simian iPSCs (siPSCs) could be used as a new source of liver cells for employment in large animal models for preclinical studies. Their results will pave the way to explore co-culture systems that could be assessed during preclinical studies, including using autologous monkey donors, for regenerative medicine purposes [10]. Rou Xiao et al. describe their genetic method for the correction of Duchenne Muscular Dystrophy (DMD) patient-derived induced pluripotent stem cells (DMD-iPSCs) via targeted exon addition and differentiating them into suitable cells for transplantation into patients. This methodology has clinical prospects for DMD gene therapy despite the large size of the *DMD* gene [11]. Sanshiro Kanazawa et al. describe a method of a co-culture system comprising mesenchymal stem/stromal cells (MSCs) and hematopoietic stem/progenitor cells (HSCs) to obtain the necessary number of MSCs useful for clinical applications due to their functional ability. Their results suggest that a hematopoietic–mesenchymal signal exists, and that the state of the HSCs is important for the stability of MSC properties [12]. Hye-Jin Hur et al. tested the hypothesis that the administration of conditioned medium from neural precursor cells (NPCs; NPC-CMs) could recapitulate the beneficial effects of NPCs transplantation. Indeed, the findings demonstrated that NPC-CM promoted functional recovery and reduced cerebral infarct and inflammation with enhanced endogenous neurogenesis, suggesting the potency of NPC-CM in stroke therapy [13].

So-Yeon Park et al. exploited the potential of extracellular vesicles (EVs), nano-sized endoplasmic reticulum particles generated and released in cells; these EVs have similar biological functions as their origin cells, act as cargo for bioactive molecules (such as proteins and genetic materials) and facilitate tissue regeneration. Specifically, EVs obtained from adipose-derived MSCs (ADMSC) have neuroprotective and neurogenesis effects, and the paper describes how ADMSC-derived EVs isolated from neurogenesis-conditioned media (differentiated EVs, dEVs) promoted the neuronal differentiation of neural progenitor cells in vitro, suggesting that dEVs are a prospective candidate for EV-based neurological disorder regeneration therapy [14]. Yu-fen Chung et al. showed that the human IL12 p40-p40 homodimer (hIL12p80) within PLA and PLGA conduits improved sciatic nerve regeneration in mice. The group of conduits with NSCs and hIL12p80 (CNI) showed the best recovery and was accompanied by increased diameter of the newly regenerated nerve by two-fold. In vitro, hIL12p80 stimulated differentiation of mouse NSCs to oligodendrocyte lineages, suggesting that hIL12p80, combined with NSCs, enhanced the functional recovery of damaged nerves in mice with sciatic nerve injury. The findings suggested the hIL12 can also be applied to amyotrophic lateral sclerosis and multiple sclerosis [15]. The study of Eleonora Maurizi et al. characterized the molecular mechanisms that regulate the proliferation of corneal endothelial cells (CEnCs) in vitro using a novel molecular marker, LAP2, which is upregulated in sub-confluent growing cultures (S) while downregulated in confluent (C) CEnCs cultures. CEnCs are quiescent in vivo and they easily undergo endothelial-to-mesenchymal transition (EnMT) in vitro [16].

Chung Ming Tse et al. describe colonoid cultures derived from Caco-2/BBe cells as a three-dimensional model (3D) of the human intestinal epithelium. This culture was utilized to study two unsolved issues of lipid rafts (LRs): the membrane aggregates enriched in ceramides that are called ceramide-rich platforms (CRPs) and the intestinal Cl^−^/HCO_3_^−^ antiporter [down-regulated in adenoma (DRA)] that is enriched in LRs [17]. The study of Thibault Canceill et al. showed that Advanced Therapeutic Medical Product (ATMP) of platelet–lysate-based fibrin hydrogel (PLFH) can serve as a proper carrier for adipose-derived mesenchymal stromal cell (ASC) transplant. The in vitro 3D model enables us to investigate PLFH biomechanical properties, cell viability, proliferation and migration sustainability, and safety [18].

Wei-Han Lin et al. generated a centimeter-scale human cardiac disk and coupled it to a hydrogel-based soft-pressure sensor based on their recently established method of 3D bioprint human pluripotent stem cells in an extracellular matrix-based bioink that allows for in situ cell expansion prior to cardiac differentiation. This, bionic-engineered testbeds can be indicators of tissue health and function and provide a unique insight into human cell responses to ablative interventions [19]. Irina Nedorubova et al. developed new gene-activated matrices based on polylactide granules (PLA) impregnated with *BMP2* polyplexes and included in chitosan hydrogel or PRP-based fibrin 3D hydrogel. When implanted into critical-size calvarial defects in rats, all matrices with *BMP2* polyplexes led to new bone formation. The most significant effect on osteoinduction was observed for the PLA/PRP matrices, suggesting that PLA granules and PRP-based fibrin hydrogel containing *BMP2* polyplexes are the most promising for future applications in bone regeneration [20].

The review of Alexandra Dobranici et al. addresses magnetic fields and nanoparticles (MNPs) that may trigger focal adhesion changes, which are further translated into the reorganization of the cytoskeleton architecture and have an impact on nuclear morphology and positioning through the activation of mechanotransduction pathways. Their review presents an updated and integrated perspective on the molecular mechanisms that govern the cellular response to magnetic cues, with a special focus on neurogenic differentiation and the possible utility of nervous TE, as well as the limitations of using magnetism for these applications [21]. Agnieszka Surowiecka et al. describe in their review the use of mesenchymal stem cells in the treatment of a burn wound and conclude that cellular therapy in burn wound treatment remains an experimental method [22]. Greta Ionela Barbulescu et al. review the knowledge on cardiac decellularized extracellular matrix (dECM), a promising approach in tissue-regenerative medicine, and give an overview on future challenges in bioengineering a human heart suitable for transplantation [23]. Juan Antonio Rojas-Murillo et al. review the biological, physical, and mechanical properties of fibrin as a biomaterial for cartilage tissue engineering and as an element to enhance the regeneration or repair of chondral lesions [24]. Huamin zhang et al. review upcoming stem cell transplantation methods for clinical application, and the results of ongoing clinical trials, to provide ideas for the clinical use of stem cell transplantation as a potential treatment for Inflammatory bowel disease (IBD), a chronic, relapsing disease that severely affects patients’ quality of life [25]. Yifang Li et al. discussed in their review the preclinical data available on the anti-fibrotic and renoprotective actions of bone-marrow-derived mesenchymal stromal cells (BM-MSCs) and the anti-fibrotic drug serelaxin (RLX), alone and when combined, as a treatment option for normotensive vs. hypertensive Chronic Kidney Disease (CKD) [26].
